# Supplementary material for: Tp63-expressing adult epithelial stem cells cross lineages boundaries revealing latent hairy skin competence
Source: Nat Commun. 2020 Nov 6;11:5645. doi: 10.1038/s41467-020-19485-3 (PMC7648065; doi:10.1038/s41467-020-19485-3)
Supplement: Supplementary file 1 — Supplementary Information [file 41467_2020_19485_MOESM1_ESM.pdf]

Supplementary information

Tp63-Expressing Adult Epithelial Stem Cells Cross Lineages Boundaries Revealing Latent Hairy Skin Competence

Claudinot et al.

Supplementary Figures 1 to 7

Supplementary Tables 1 to 8

Additional References

Supplementary Figure 1

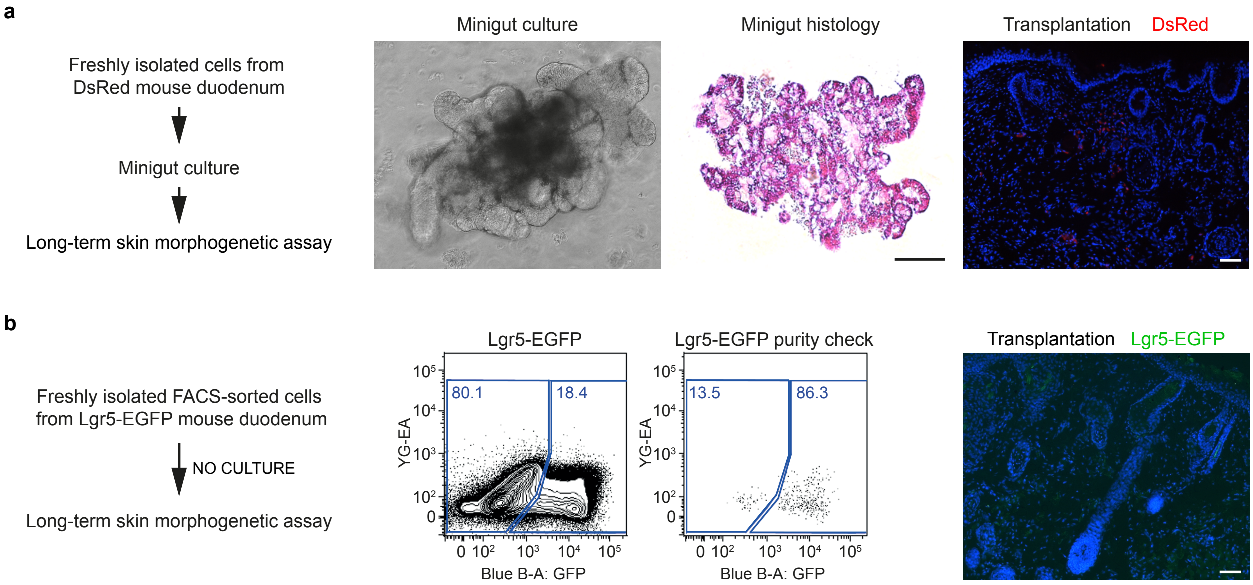

Supplementary Figure 1. Stem cells from the duodenum of the mouse do not respond to a skin microenvironment. **a** From left to right: schematic representation of the experimental design to assess the skin-forming ability of cells isolated from minigut cultures derived from epithelial cells isolated from the duodenum of B6.Cg-Tg(CAG-DsRed*MST)1Nagy/J mice; phase contrast microscopy of a minigut obtained from DsRed duodenal cells; histology of a minigut; no engraftment of the transplanted DsRed cells after 40 days of transplantation (DsRed immunocytochemistry); nuclei (blue) were counterstained with Hoechst 33342; n= 3 mice. **b** From left to right: schematic representation of the experimental design to assess the skin-forming ability of Lgr5-EGP positive cells isolated from B6.129P2-Lgr5tm1(cre/ERT2)Cle/J mice; results of the FACS-sorting of the Lgr5-EGFP positive duodenal cells, Lgr5-EGFP purity check plot; no engraftment of the Lgr5-EGFP sorted cells after 39 days of transplantation (EGFP immunocytochemistry); nuclei (blue) were counterstained with Hoechst 33342; n= 8 mice. Bars: 100μm.

Supplementary Figure 2

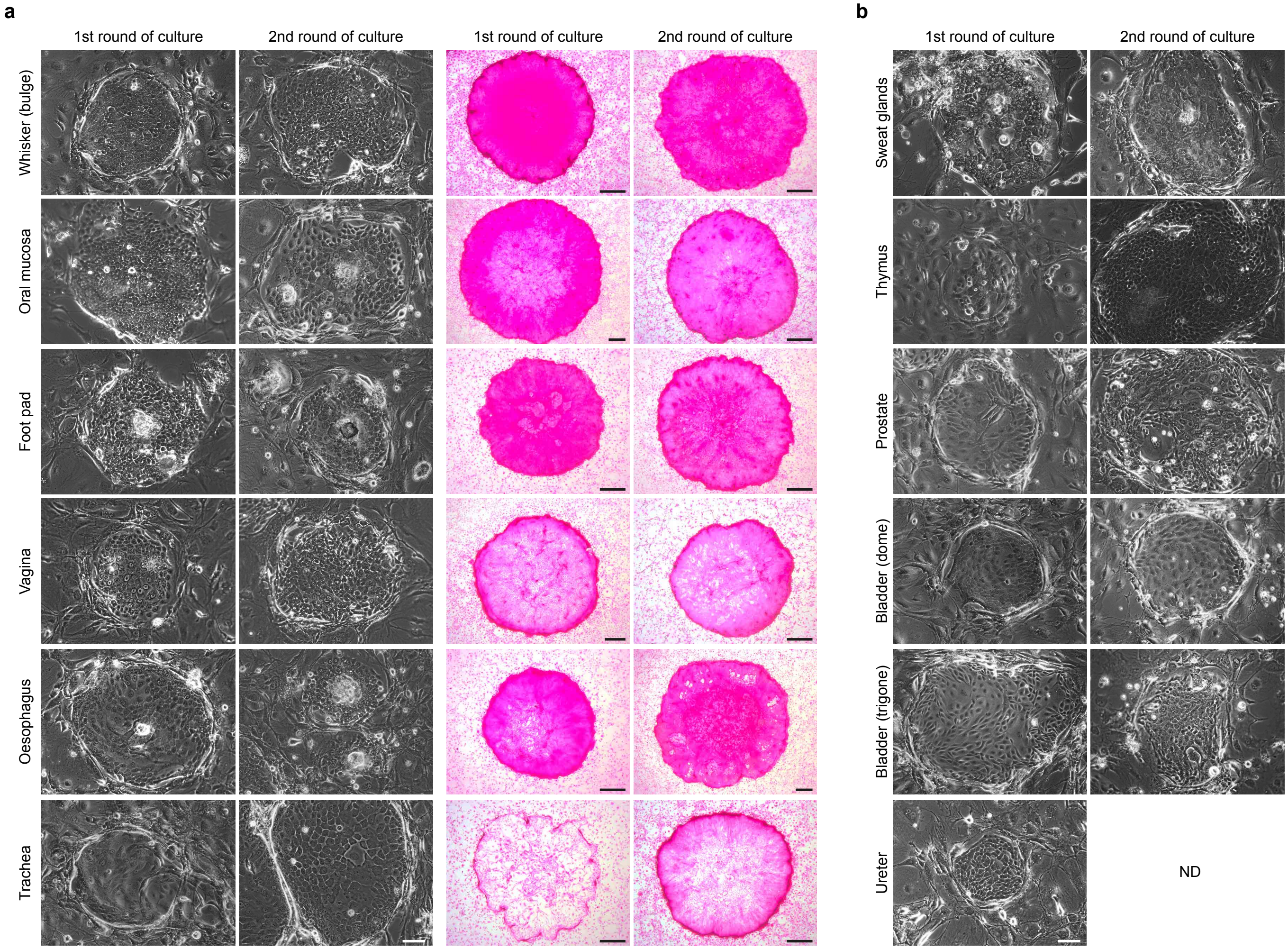

Supplementary Figure 2. Appearance of colonies formed by Tp63-expressing epithelial stem cells. Cells were cultured before their transplantation into a newborn mouse skin microenvironment (1st round of culture) and after recovery from the transplants (2nd round of culture); 4 independent experiments for each cell type. **a** Left panel: phase contrast microscopy of 3-8 days old progressively growing; bars: 100µm. Right panel: 10-12 days old colonies after formaldehyde fixation and Rhodamine B staining (red). Bars: 1mm. **b** Phase contrast microscopy of 3-8 days old progressively growing colonies formed by other Tp63-expressing epithelial stem cells. ND: no data. Bars: 100µm.

Supplementary Figure 3

a

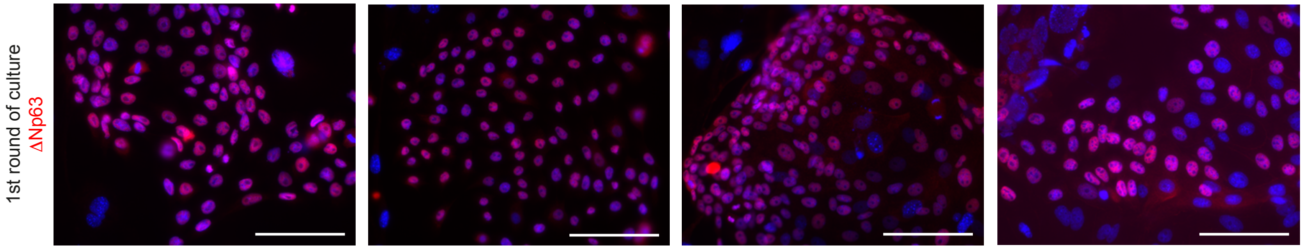

b

Tissue (EGFP rat)	1st round of culture	1st transplantation						2nd round of culture	2nd transplantation					
	Passage	Number of transplantation	Presence of GFP+ cells (%)	HF	Skin SG	Epid.	Others	Passage	Number of transplantation	Presence of GFP+ cells (%)	HF	Skin SG	Epid.	Others
Bladder (dome)	II	3	100	+	+	+	Cysts	III	2	50	+	+	-	-
Bladder (trigone)	II	3	33	+	+	+	Cysts	III	2	50	+	+	-	-
Prostate	II	2	50	+	+	+	Cysts	III-IV	3	0	-	-	-	-
Whisker (bulge)	XI-XIV	20	75	+	+	+	-	III	2	100	+	+	-	-
Oral mucosa	III-IV	13	61.54	+	+	-	-	III	6	66.66	+	+	+/-	-
Foot pad	II-IV	7	14.3	+	+	-	-	IV	5	20	+	+	-	-
Sweat glands	V	5	80	+	+	-	-	III-IV	3	33.3	+	+	-	-
Vagina	III-IV	8	50	+	+	-	-	III	10	20	+	+	-	-
Oesophagus	III-IV	7	57.15	+	+	-	-	IV-V	19	26.31	+	+	+/-	Cysts
Trachea	I-IV	8	25	-	-	+	-	III	17	47.06	-	-	+	-
Duodenum	ND	11	0	-	-	-	-	ND	ND	ND	ND	ND	ND	ND

c

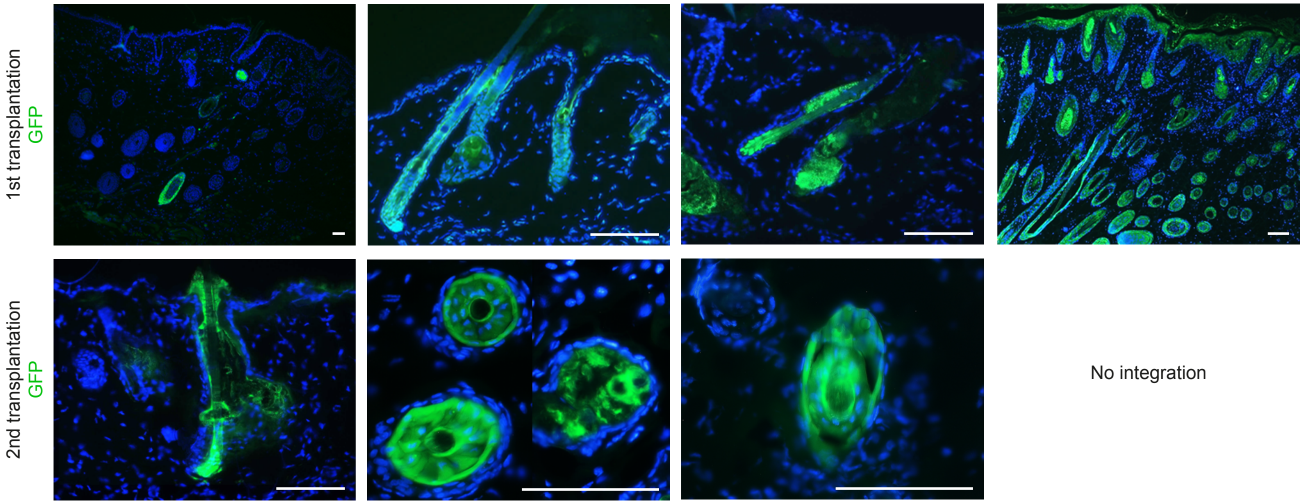

d

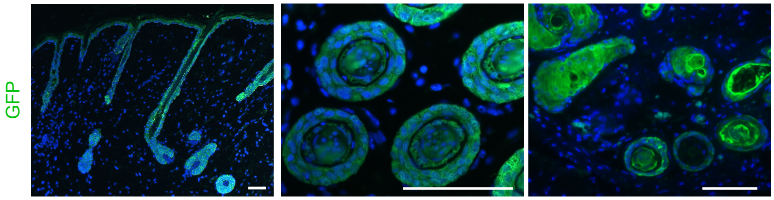

e

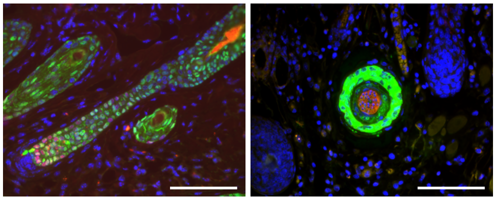

Supplementary Figure 3. Skin forming-ability of Tp63-expressing cells. a Representative experiment showing that cells isolated from various epithelial tissues expressed ΔNp63 isoforms in culture (immunocytochemistry, red nuclear staining); nuclei (blue) were counterstained with Hoechst 33342; bars: 100µm. **b** Summary of all transplantation experiments. A schematic representation of the experimental design is shown in fig. 1a; HF: hair follicles, SG: sebaceous glands, Epid: epidermis, ND: no data. Participation of the transplanted cultured stem cells to the formation of hair follicles and sebaceous glands: + frequent, +/- rare, - none. **c** Presence of EGFP positive rat cells in hair follicles (EGFP immunocytochemistry). Upper panel: 1st transplantation; oral mucosa: day 100, n= 13 mice; footpad: day 99, n= 7 mice; vagina: day 84, n= 8 mice, prostate: day 103, n= 2 mice. Lower panel: 2nd transplantation; oral mucosa: day 100, n= 6 mice; footpad: day 113, n= 5 mice; vagina: day 99, n= 10 mice, prostate: no integration, n= 3 mice. Bars: 100µm. **d** Presence of EGFP positive rat cells in hair follicles; first transplantation: bladder (trigone): day 238, n= 3 mice; prostate: day 238, n= 2 mice; second transplantation: oesophagus: day 100, n= 19 mice. Bars: 100µm. **e** Representative immunohistochemistry showing co-expression of EGFP (green), LHX2 or CK-31 (red) in hair follicles formed by epithelial cells cultured from the prostate of an EGFP rat; first transplantation, day 103 (left) and day 238 (right). Nuclei (blue) were counterstained with Hoechst 33342. Bars: 100µm.

Supplementary Figure 4

a

Tissue of interest	Number of tested clones	Number of transplantations	Presence of transplanted cells (%)	Skin morphogenesis			Others
				HF	SG	Epid.	
Whisker (euploid)	17	56	60.71	+	+	+	-
Whisker (aneuploid)	2	11	0	-	-	-	-
Foot pad (aneuploid)	1	4	0	-	-	-	-
Vagina (aneuploid)	6	27	0	-	-	-	-
Oesophagus (aneuploid)	4	18	0	-	-	-	-

b

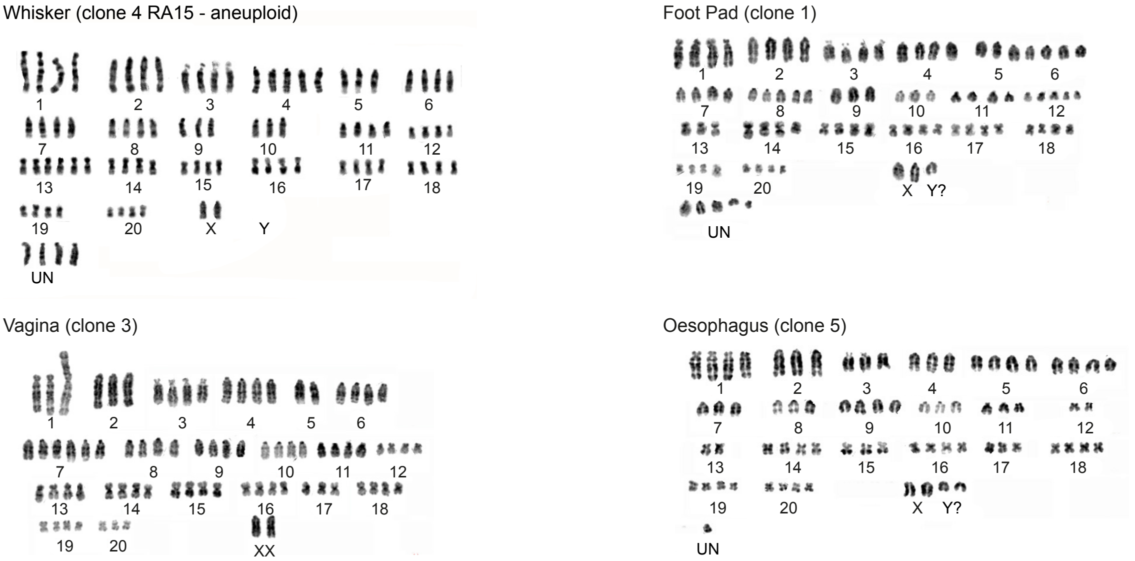

c

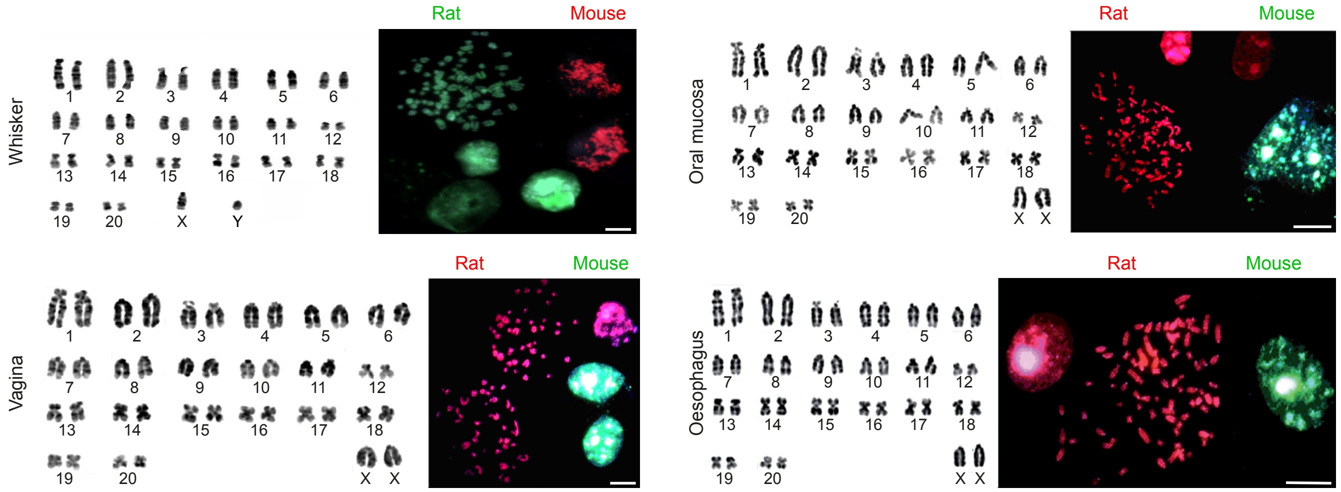

Supplementary Figure 4. Diploidy is mandatory for permanent engraftment. a Aneuploid clones derived from cultures of clonogenic epithelial cells isolated from rat whisker follicle, footpad, vagina and oesophagus epithelial cells do not engraft. **b** Karyotype of aneuploid cells. **c** FISH demonstrating that donor rat cells did not fuse with resident mouse cells. The experiments were performed on EGFP cells recovered and cultured at the end of the 1st round of transplantation. Rat chromosomes are labelled in red and mouse chromosomes in green, except for whisker cells in which the colours are inverted. Bars= 20 µm.

Supplementary Figure 5

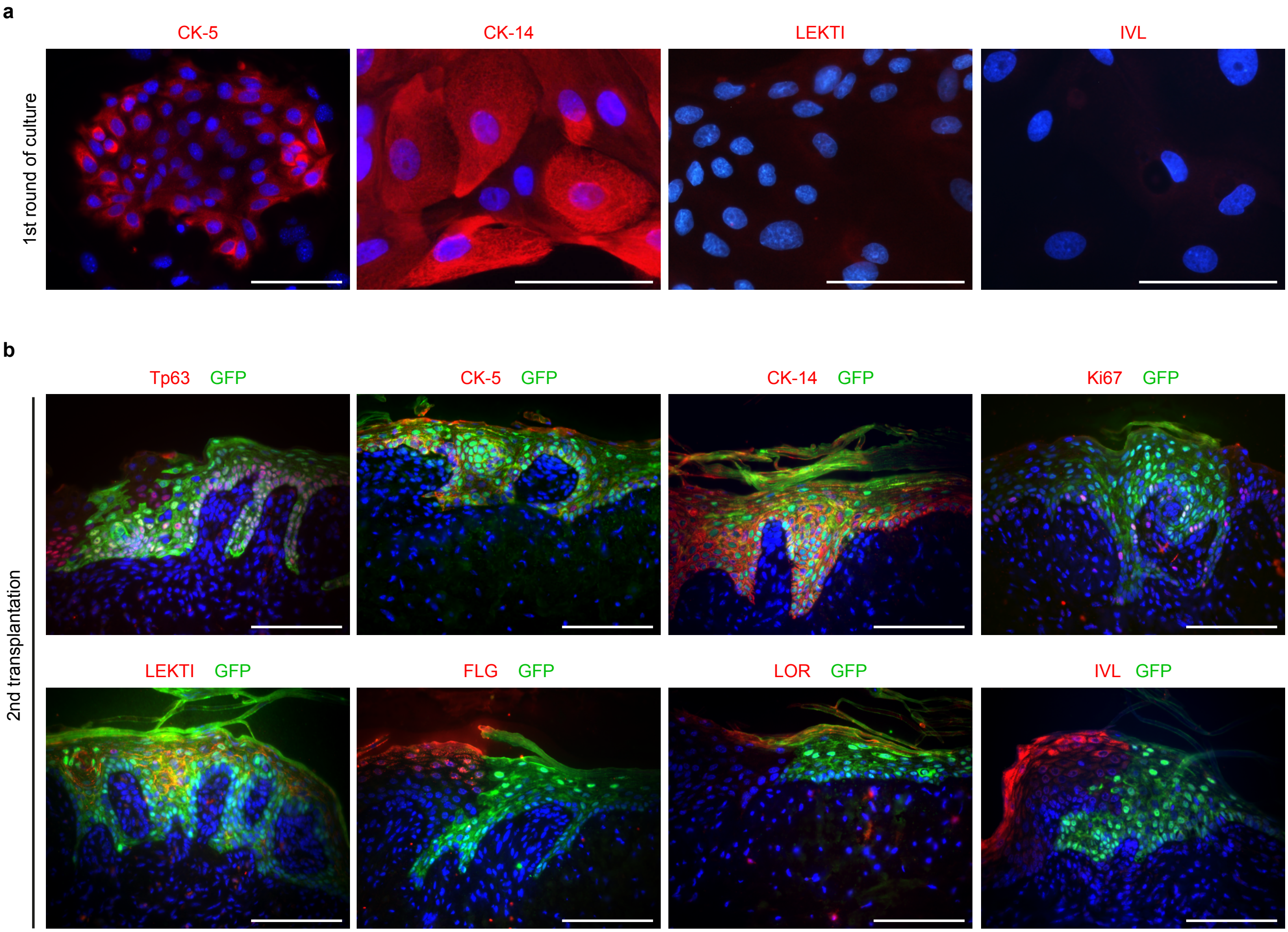

Supplementary Figure 5. Expression of markers of squamous differentiation in transplanted tracheal cells. a Epithelial cells cultured from the trachea of the rat express markers of basal cells of stratified epithelia (CK5, CK14) but not squamous differentiation markers like involucrin (IVL) or LEKTI. Bars: 100µm. Representative of 2 experiments. **b** Representative immunocytochemistry experiment showing that EGFP tracheal cells (green) transplanted into the skin of a newborn wild type mouse expressed Tp63, cytokeratin-5 (CK-5), cytokeratin-14 (CK-14), Ki67 but not squamous differentiation markers like LEKTI, filaggrin (FLG), loricrin (LOR) or involucrin (IVL). Note that the later markers are expressed in the mouse epidermis adjacent to the transplant. Bars: 100µm.

Supplementary Figure 6

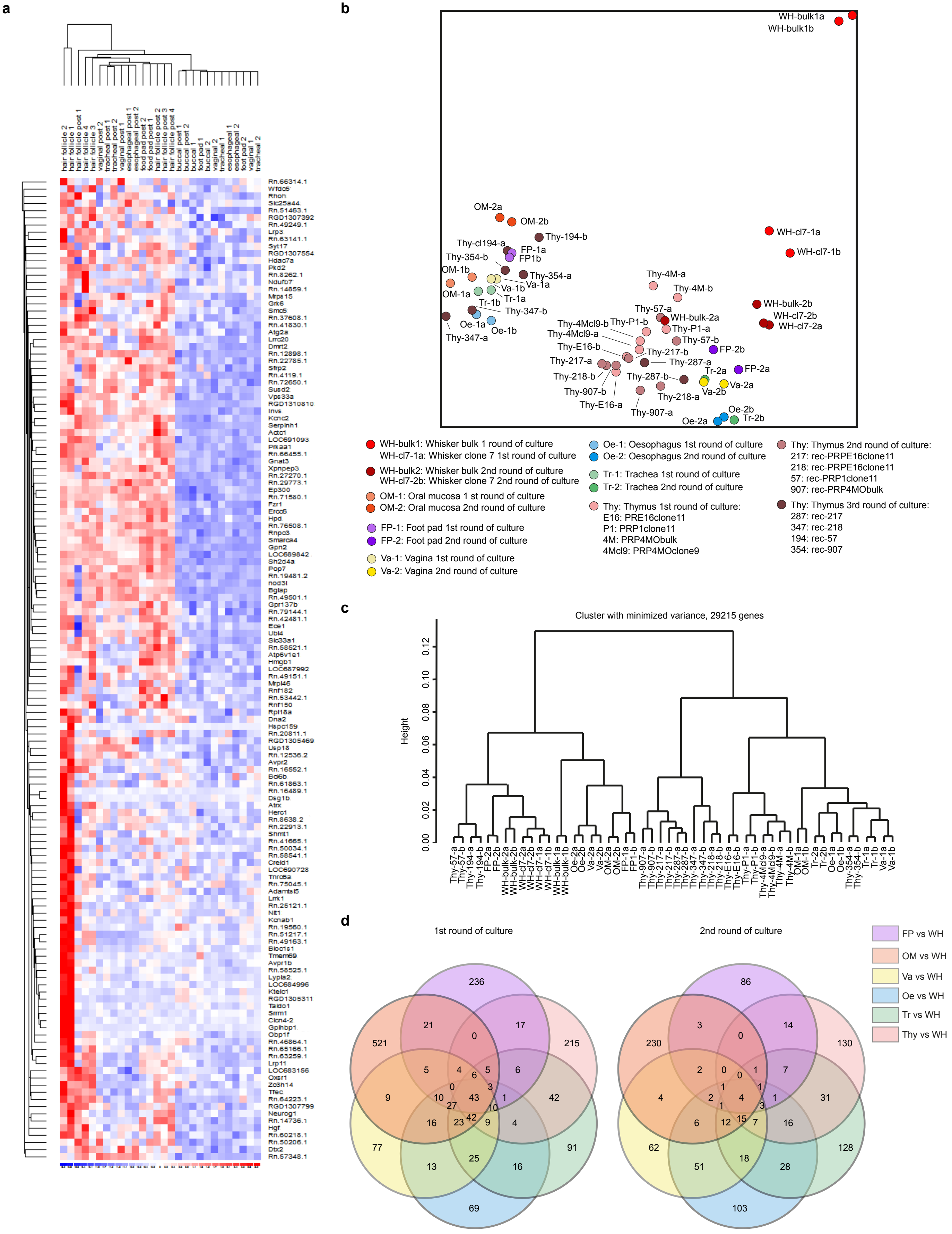

Supplementary Figure 6. Microarray analysis. A microarray analysis was performed on epithelial stem cells cultured from whisker (WH), oral mucosa (OM), footpad (FP), vagina (VA), oesophagus (Oe), thymic (Thy) and trachea (TR) before they were transplanted into a newborn mouse skin microenvironment (1st round) and after they were recovered from the transplants (2nd round recovered). Cells were FACS sorted on EGFP expression before total RNA was extracted. **a** The list of all 137genes specifically up-regulated in hair follicle versus all other non-transplanted tissues (fold-change = 1.5) was used to define a "hair follicle blueprint". The expression of these "blueprint" genes was then analyzed in cells before and after transplantation (post). **b** Principal component analysis (PCA) of the full transcriptomes. **c** Expression clustering analysis. **d** Venn diagram showing the number of up- and down-regulated genes, fold-change >2 for each tissue in comparison to hair follicle. Adjusted p-value <0.05.

Supplemental Figure 7

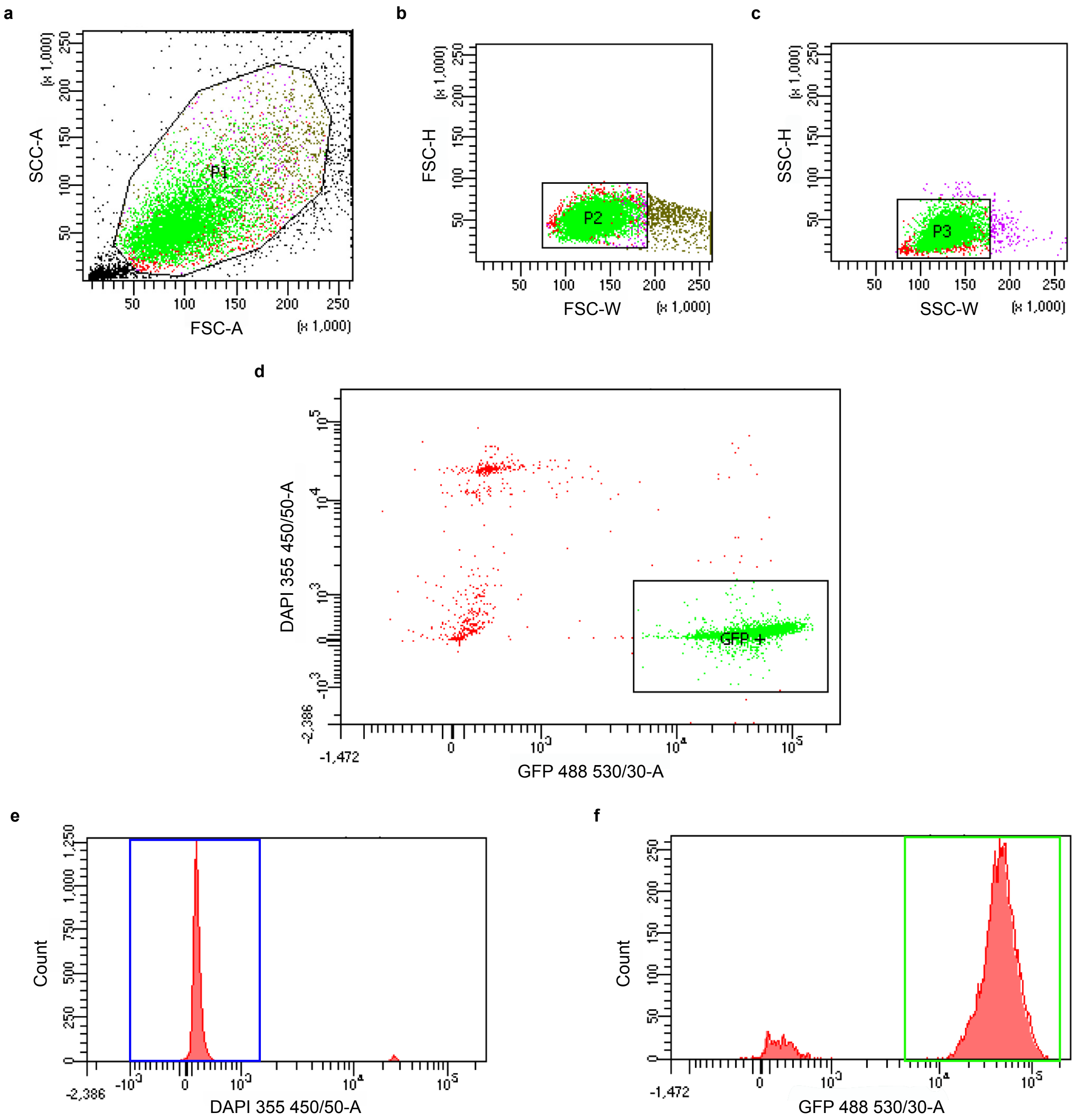

Supplementary Figure 7. Gating for Fluorescent Activated Cell Sorting of cultured EGFP-positive rat cells for RNA-Seq analysis. The gating strategy was identical for all samples. **a** Elimination of the debris from the total cell suspension. **b** Elimination of doublets and **c** selection of singlets. **d** Selection of living EGFP-positive cells on DAPI-dye exclusion and EGFP expression. **e** Histogram of DAPI expression. Gates are represented in a blue rectangle. **f** Intensity distribution of EGFP expression in the selected population. Gates are represented by a green rectangle.

Supplementary Table 1.

Tissue	Epithelial type	Embryonic origin	Tp63	References
Skin	Stratified squamous keratinized	Ectoderm	+	(1-3)
Foot pad	Stratified squamous keratinized	Ectoderm	+	(4)
Sweat glands	Simple and stratified cuboidal	Ectoderm	+	(3)
Oral mucosa	Stratified squamous non-keratinized	Ectoderm	+	(2, 5)
Ocular surface	Stratified squamous non-keratinized	Ectoderm	+	(6)
Anal canal	Stratified squamous non-keratinized	Ectoderm	+	(7)
Vagina	Stratified squamous non-keratinized	Mesoderm	+	(1, 8)
Oesophagus	Stratified squamous non-keratinized	Endoderm	+	(3, 9)
Thymus	Reticular	Endoderm	+	(10, 11)
Bladder	Transitional	Endoderm	+	(3, 7)
Prostate	Pseudostratified columnar	Endoderm	+	(1, 7)
Trachea	Pseudostratified	Endoderm	+	(5, 9)
Ureter	Transitional	Mesoderm	+	(12)
Duodenum	Simple	Endoderm	-	(3)

Supplementary Table 1. Tp63 expression in mammalian epithelia. The transcription factor Tp63 is expressed in stratified and glandular epithelia but not in simple epithelia.

Supplementary Table 2.

Tissue	Aberrant skin appendages		Epidermoid/ dermoid cyst	Squamous metaplasia	Squamous cell carcinoma	References
	Human	Other mammals				
Skin	+ (HF, SG)	+ (HF, SG)	+	-	+	(13-15)
Foot pad	Rare (HF-SG)	No data	+	-	+	(16-19)
Sweat glands	No data	No data	+	+	Rare	(20-22)
Oral mucosa	Rare (HF-SG)	No data	Rare	+	+	(23-27)
Ocular surface	+ (HF)	+	Rare	+	+	(28-32)
Anal canal	No data	No data	Glandular	+	+	(33-35)
Vagina	Rare (HF-SG)	No data	+	+	+	(36-39)
Oesophagus	Rare (SG)	No data	Rare	+/Barrett/ sebaceous	+	(40-46)
Thymus	Rare (SG)	No data	Rare	No data	Rare	(47-49)
Bladder	No data	No data	Rare	+	+	(50-52)
Prostate	No data	No data	Rare	+	Rare	(53-55)
Trachea	No data	No data	Glandular	+	+	(56-58)
Ureter	No data	No data	Very rare	Very rare	+	(59-61)
Duodenum	No data	No data	No data	No data	Very rare	(62)

Supplementary Table 2. Aberrant hair follicles and dermoid cysts in non-hairy mammalian tissues. Data from the literature reporting the presence of ectopic skin appendages, dermoid cysts, squamous metaplasia or squamous cell carcinoma in various non-hairy epithelial tissues.

Supplementary Table 3

ID	Gene.Symbol	1st round of culture<br>Oral m. vs HF	Foot p. vs HF	Vagina vs HF	Oeso. vs HF	Trachea vs HF	Thymus vs HF
10774605	---	-3.307773043	-3.387957437	-3.22021441	-3.164114374	-3.279642929	-2.607791452
10719530	Apoe	-3.43050002	-2.915881065	-4.649239914	-4.192336078	-3.993252711	-3.097815559
10775866	Areg	3.01557763	2.266357378	2.707496316	2.81303865	2.817644062	2.413821928
10708954	Arrb1	15.26931434	18.10463109	18.3949822	11.01916405	14.90501451	3.024268803
10853995	Asb15	-2.535219875	-2.129924623	-3.006562345	-2.629520738	-2.728377698	-2.213816854
10851952	B4galt5	3.026544891	2.349566743	3.530937415	2.419103927	5.593866214	2.932438293
10774596	Bcl11a	-5.288661907	-3.678873961	-5.386175543	-4.937820012	-4.758885522	-3.380316306
10775573	Bmp3	5.227876735	4.291974858	2.257142691	11.33847434	6.272671997	3.309342532
10874811	C1qdc2	-8.161411868	-8.933510771	-10.84268572	-13.20291194	-11.35269372	-14.3757356
10887802	Cdca7l	-3.245717643	-2.286529449	-2.319678715	-2.172331836	-2.255128277	-2.663685261
10901409	Chst11	-8.178059872	-4.904041287	-3.907733618	-10.49792743	-3.936830572	-2.327275693
10836588	Dhrs9	2.763692779	7.687019083	6.523402966	3.866028438	4.470689188	6.618880046
10716454	Emx2	2.971821059	2.326400928	6.970328985	3.400313464	3.779576919	4.269568803
10896661	Fam83a	3.364603497	3.819356267	2.409574655	3.184398747	-7.183908625	-3.969059322
10848416	Fsip1	-2.917285839	-2.432041337	-2.945804626	-2.3907483	-2.219490833	-2.140090787
10796220	Gata3	-6.03145992	-3.463459371	-4.196087464	-6.179708341	-5.895727113	-3.713553531
10818291	Gstm5	-3.237328462	-6.173832105	-4.26368912	-3.588127735	-3.655507597	-2.131585058
10863430	Hk2	2.837072218	2.096969496	3.209276016	2.524696337	2.775546522	2.808297204
10924245	Il8rb	19.05514921	19.95863983	23.58435107	13.54094011	5.466833739	7.509600315
10892835	Itgb8	-7.23002432	-6.296405692	-5.632488645	-5.325740005	-6.312611651	-7.38352887
10925405	Klhl30	4.221384891	2.101073127	2.499201761	2.992839423	2.144888087	2.218941488
10838354	Lgr4	-5.271618616	-6.921290218	-4.571133647	-6.060179389	-4.731784925	-4.183545498
10835958	Lhx2	-2.94850318	-2.197622749	-4.091777649	-2.519018557	-3.271175698	-3.292495286
10823949	Lrat	-17.51577448	-11.58228803	-7.404466929	-8.847254811	-16.18441008	-18.26598628
10859392	Mgst1	-14.62852977	-4.292246333	-14.76432377	-6.015440449	-5.503015434	-10.76339462
10724042	P2ry2	4.344094914	3.593802496	3.482960533	3.614372314	3.499305508	2.442632588
10937362	Pak3	-5.52227602	-5.379160367	-5.322920429	-6.594432454	-5.054454857	-2.299646925
10826371	Palmd	-16.67325357	-9.19041335	-16.23356832	-13.7352451	-16.91507595	-6.31812454
10884688	Pax9	4.452888154	2.009648065	2.50926767	6.380364632	4.203435323	6.525038888
10895406	Phlda1	2.608103278	2.692350057	3.382596283	2.255412004	2.668392701	2.528196326
10723576	Prss23	-7.73719562	-7.418025617	-6.541512509	-14.03300672	-3.570777524	-9.765646122
10796440	Pter	-2.766719959	-2.258748472	-2.687395442	-2.576643827	-2.508731591	-2.812136015
10844223	Ptges	8.655891258	2.824778753	5.875034709	4.000321287	4.898918786	5.033513819
10835817	Ptgs1	19.24764121	29.44241791	39.75014588	27.73018334	23.78790394	23.71438008
10864874	Rassf4	-4.663751762	-4.211557899	-6.70706926	-4.823515492	-5.656152428	-5.944809686
10876730	RGD1307218	-3.079695044	-6.252950082	-4.581147455	-4.698033498	-4.950624409	-3.422588864
10804292	RGD1563060	-3.420893345	-2.814114686	-3.656495598	-2.975096831	-4.168002448	-2.663101425
10754592	RGD1564641	-2.972467426	-3.822077415	-6.695839747	-16.99291557	-10.1912351	-6.007010248
10804415	Sema6a	2.344371575	2.113908234	3.325910278	2.917132163	4.115555721	3.08672215
10866271	Styk1	2.405753162	2.738677001	3.966122189	2.357333907	4.156761936	5.602061222
10891303	Tgfb3	-6.364638725	-7.419341388	-2.624817513	-7.2767393	-2.531139681	-4.740943665
10880074	Tinagl1	3.440420126	2.298439593	2.479681367	2.74458829	2.353821224	3.072775253
10873021	Wnt4	2.084827653	3.050620302	3.159913628	3.580807902	3.337055371	2.294758369

Supplementary Table 3. List of the genes in common in the microarray analysis. Up- and down-regulated genes in common in different non-hairy Tp63-expressing epithelial stem cells cultured before and after transplantation into the skin of newborn wild type mouse compared to that of multipotent stem cells of the whisker follicle. Microarray analysis. Fold change > 2, adjusted p-value <0.05.

Supplementary Table 4.

Comparison1	Comparison2	N_Genes_Comparison1	N_Genes_Comparison2	N_Genes_Overlap	N_Genes_Total	Pvalue_Hyperg_Over	Pvalue_Hyperg_Under
Va vs WH	BL vs WH	2528	4215	1794	13258	0	1
Va vs WH	Thy vs WH	2528	3071	1671	13258	0	1
Va vs WH	Tr vs WH	2528	3775	1845	13258	0	1
BL vs WH	Thy vs WH	4215	3071	2093	13258	0	1
BL vs WH	Tr vs WH	4215	3775	2875	13258	0	1
Thy vs WH	Tr vs WH	3071	3775	2077	13258	0	1

Key

Comparison1	Name of comparison 1
Comparison2	Name of comparison 2
N_Genes_Comparison1	Number of genes in comparison 1
N_Genes_Comparison2	Number of genes in comparison 2
N_Genes_Overlap	Number of overlapping genes
N_Genes_Total	Total number of expressed genes (background)
Pvalue_Hyperg_Over	Hypergeometric p-value for over-representation
Pvalue_Hyperg_Under	Hypergeometric p-value for under-representation

Supplementary Table 4. **Results of the hypergeometric test before transplantation.**

Supplementary Table 5

Comparison1	Comparison2	N_Genes_Comparison1	N_Genes_Comparison2	N_Genes_Overlap	N_Genes_Total	Pvalue_Hyperg_Over	Pvalue_Hyperg_Under
Va vs WH	BL vs WH	2528	4215	1794	13258	0	1
Va vs WH	Thy vs WH	2528	3071	1671	13258	0	1
Va vs WH	Tr vs WH	2528	3775	1845	13258	0	1
BL vs WH	Thy vs WH	4215	3071	2093	13258	0	1
BL vs WH	Tr vs WH	4215	3775	2875	13258	0	1
Thy vs WH	Tr vs WH	3071	3775	2077	13258	0	1

Key

Comparison1	Name of comparison 1
Comparison2	Name of comparison 2
N_Genes_Comparison1	Number of genes in comparison 1
N_Genes_Comparison2	Number of genes in comparison 2
N_Genes_Overlap	Number of overlapping genes
N_Genes_Total	Total number of expressed genes (background)
Pvalue_Hyperg_Over	Hypergeometric p-value for over-representation
Pvalue_Hyperg_Under	Hypergeometric p-value for under-representation

Supplementary Table 5. Results of the hypergeometric test after transplantation.

Supplementary Table 6

Tissue of origin (Rosa26-LacZ)	Embryonic origin	Epithelial classification	Number of transplanted mice	Participation in hairy skin (%)
Bulge (whisker)	Ectoderm	Pluristratified	12	58.3
Bulb (whisker)	Ectoderm	Pluristratified	2	50
Central cornea	Ectoderm	Pluristratified	8	50
Limbus	Ectoderm	Pluristratified	39	33.3
Conjunctiva	Ectoderm	Pluristratified	6	66.6
Eyelid	Ectoderm	Pluristratified	2	100
Oral mucosa	Ectoderm	Pluristratified	10	50
Soft palate	Ectoderm	Pluristratified	8	50
Foot pad	Ectoderm	Pluristratified	19	52.6
Anal mucosa	Ectoderm	Pluristratified	8	37.5
Vagina	Mesoderm	Pluristratified	23	65.2
Oesophagus	Endoderm	Pluristratified	19	63.2
Trachea	Endoderm	Pseudostratified	12	0
Bladder	Endoderm	Transitional	8	0
Thymus	Endoderm	Reticular	3	0
Uterus	Endoderm	Simple	4	0
Duodenum	Endoderm	Simple	12	0
Colon	Endoderm	Simple	9	0

Supplementary Table 6. Results of the transplantation of non-cultured epithelial tissues. Full-thickness biopsies of various epithelial tissues obtained from Rosa26-*LacZ* mice were transplanted into the back skin of newborn wild type mice to evaluate their response to a skin microenvironment.

Supplementary Table 7. List of Antibodies

	Antigen	Specie	Clone	Concentration	Catalog #	Provider
Primary Antibody	Cytokeratin 4	Mouse	6B10	1 :200	NCL-CK4	Novocastra
	Cytokeratin 5	Rabbit	-	1 :1000	ab24647	Abcam
	Cytokeratin 8/18	Guinea Pig	-	1 :100	GP-11	Progen
	Cytokeratin 14	Rabbit	-	1 :1000	PRB-155P-100	Covance
	Cytokeratin 15	Mouse	LHK15	1 :100	MA5-11344	ThermoFisher
	Cytokeratin 31	Guinea Pig	-	1 :200	GP-HHA1	Progen
	ΔNp63	Rabbit	-	1 :500	619001	Biolegend
	DsRed	Rabbit	-	1 :100	GTX59862	Genetex
	Filaggrin	Rabbit	-	1 :400	PRB-417P	Covance
	GFP	Chicken	-	1 :400	ab13970	Abcam
	Involucrin	Mouse	SY5	1 :100	MA5-11803	ThermoFisher
	Ki67	Mouse	B56	1 :200	556003	BD Biosciences
	LEKTI	Mouse	1C11G6	1 :100	39-0500	Invitrogen
	Lhx2	Rabbit	EPR20449	1 :500	ab184337	Abcam
	Loricrin	Rabbit	-	1 :200	GTX 116013	Genetex
	Sox9	Rabbit	EPR14335-78	1 :500	ab185966	Abcam
	Tp63	Mouse	4A4	1 :500	M7247	Dako
				1 :500	GTX23239	Genetex
				1 :500	ab735	Abcam
Secondary Antibody	Anti-mouse Alexa 568	Goat	-	1 :500	A11004	Molecular Probes
	Anti-Rabbit Alexa 568	Goat	-	1 :500	A11011	Molecular Probes
	Anti-Mouse Alexa 568	Goat	-	1 :500	A11031	Molecular Probes
	Anti-Chicken Alexa 488	Goat	-	1 :500	A11039	Molecular Probes
	Anti-Guinea Pig Alexa 568	Goat	-	1 :500	A11075	Molecular Probes
	Anti-Chicken Alexa 488	Goat	-	1 :500	ab150169	Abcam

Supplementary Table 8. List of Primers

Gene	Species	Assay ID	Catalog #	Dye
Dkk1	Rat	Rn01501537_m1	4448892	FAM-MGB
Dkk3	Rat	Rn00593415_m1	4448892	FAM-MGB
ΔNp63	Rat	Rn01404786_m1	4448892	FAM-MGB
Foxn1	Rat	Rn01460456_m1	4448892	FAM-MGB
Gata3	Rat	Rn00484683_m1	4448892	FAM-MGB
Hr	Rat	Rn00577605_m1	4448892	FAM-MGB
Jag1	Rat	Rn00569647_m1	4448892	FAM-MGB
Lef1	Rat	Rn01639120_m1	4448892	FAM-MGB
Lhx2	Rat	Rn01449595_m1	4448892	FAM-MGB
Notch1	Rat	Rn01758633_m1	4448892	FAM-MGB
Sdha	Rat	Rn00590475_m1	4453320	FAM-MGB
TAp63	Rat	Rn00570095_m1	4448892	FAM-MGB
Tbp	Rat	Rn01455646_m1	4453320	FAM-MGB
Tp63	Rat	Rn00693067_m1	4448892	FAM-MGB
Tubb3	Rat	Rn01431594_m1	4448892	FAM-MGB
Wnt3	Rat	Rn01471585_m1	4448892	FAM-MGB
Wnt3a	Rat	Rn01470643_m1	4331182	FAM-MGB
Wnt5a	Rat	Rn01402000_m1	4448892	FAM-MGB
Wnt10a	Rat	Rn01401164_m1	4448892	FAM-MGB

Additional References

1. Yang, A. *et al.* p63 is essential for regenerative proliferation in limb, craniofacial and epithelial development. *Nature* **398**, 714-718 (1999).
2. Mills, A.A. *et al.* p63 is a p53 homologue required for limb and epidermal morphogenesis. *Nature* **398**, 708-713 (1999).
3. Di Como, C.J. *et al.* p63 expression profiles in human normal and tumor tissues. *Clin Cancer Res.* **8**, 494-501 (2002).
4. Kanitakis, J. *et al.* Circumscribed palmo-plantar hypokeratosis: a disease of desquamation? Immunohistological study of five cases and literature review. *J Eur Acad Dermatol Venereol* **25**, 296-301 (2011).
5. Dellavalle, R.P. *et al.* CUSP/p63 expression in rat and human tissues. *J Dermatol Sci* **27**, 82-87 (2001).
6. Pellegrini, G. *et al.* p63 identifies keratinocyte stem cells. *Proc Natl Acad Sci U S A* **98**, 3156-3161 (2001).
7. Pignon, J.C. *et al.* p63-expressing cells are the stem cells of developing prostate, bladder, and colorectal epithelia. *Proc Natl Acad Sci U S A* **110**, 8105-8110 (2013).
8. Kurita, T., Cunha, G.R., Robboy, S.J., Mills, A.A. & Medina, R.T. Differential expression of p63 isoforms in female reproductive organs. *Mech Dev* **122**, 1043-1055 (2005).
9. Daniely, Y. *et al.* Critical role of p63 in the development of a normal esophageal and tracheobronchial epithelium. *Am J Physiol Cell Physiol* **287**, C171-181 (2004).
10. Senoo, M., Pinto, F., Crum, C.P. & McKeon, F. p63 Is essential for the proliferative potential of stem cells in stratified epithelia. *Cell* **129**, 523-536 (2007).
11. Candi, E. *et al.* DeltaNp63 regulates thymic development through enhanced expression of FgfR2 and Jag2. *Proc Natl Acad Sci U S A* **104**, 11999-12004 (2007).
12. Feil, G. *et al.* Immunoreactivity of p63 in monolayered and in vitro stratified human urothelial cell cultures compared with native urothelial tissue. *Eur Urol* **53**, 1066-1072 (2008).
13. Grimalt, R. & Gelmetti, C. Eruptive vellus hair cysts: case report and review of the literature. *Pediatr Dermatol* **9**, 98-102 (1992).
14. Takeshita, T., Takeshita, H. & Irie, K. Eruptive vellus hair cyst and epidermoid cyst in a patient with pachyonychia congenita. *J Dermatol* **27**, 655-657 (2000).
15. Lapouge, G. *et al.* Identifying the cellular origin of squamous skin tumors. *Proc Natl Acad Sci U S A* **108**, 7431-7436 (2011).
16. Mehregan, A.H. & Coskey, R.J. Pigmented nevi of sole. A report of two cases with histologic evidence of hair follicle formation. *Arch Dermatol* **106**, 886-887 (1972).
17. Ohnishi, T. & Watanabe, S. Immunohistochemical observation of cytokeratins in keratinous cysts including plantar epidermoid cyst. *J Cutan Pathol* **26**, 424-429 (1999).
18. Gomi, M., Naito, K. & Obayashi, O. A large epidermoid cyst developing in the palm: A case report. *Int J Surg Case Rep* **4**, 773-777 (2013).
19. Weinrauch, L., Peled, I., Goldberg, L.H. & Wexler, M.R. Squamous cell carcinoma of the palm. *Dermatologica* **166**, 89-91 (1983).
20. Egawa, K., Egawa, N. & Honda, Y. Human papillomavirus-associated plantar epidermoid cyst related to epidermoid metaplasia of the eccrine duct epithelium: a combined histological, immunohistochemical, DNA-DNA in situ hybridization and three-dimensional reconstruction analysis. *Br J Dermatol* **152**, 961-967 (2005).
21. Dauden, E., Porras, J.I., Buezo, G.F. & Garcia-Diez, A. Eccrine squamous syringometaplasia and cytomegalovirus. *Am J Dermatopathol* **22**, 559-561 (2000).

22. Blake, P.W., Bradford, P.T., Devesa, S.S. & Toro, J.R. Cutaneous appendageal carcinoma incidence and survival patterns in the United States: a population-based study. *Arch Dermatol* **146**, 625-632 (2010).
23. Chi, A.C., Mapes, I.L., Javed, T. & Neville, B.W. Epidermal choristoma of the oral cavity: report of 2 cases of an extremely rare entity. *J Oral Maxillofac Surg* **68**, 451-455 (2010).
24. Kini, Y.K., Kharkar, V.R., Rudagi, B.M. & Kalburge, J.V. An unusual occurrence of epidermoid cyst in the buccal mucosa: a case report with review of literature. *J Maxillofac Oral Surg* **12**, 90-93 (2013).
25. Klein-Szanto, A.J., Banoczy, J. & Schroeder, H.E. Metaplastic conversion of the differentiation pattern in oral epithelia affected by leukoplakia simplex. A stereologic study. *Pathol Eur* **11**, 189-210 (1976).
26. Buckley, P., Hulse, E.V. & Keep, B.M. An inbred strain of rats with a high incidence of squamous-cell carcinomas of the mouth. *Br J Cancer* **41**, 295-301 (1980).
27. Johnson, N.W., Jayasekara, P. & Amarasinghe, A.A. Squamous cell carcinoma and precursor lesions of the oral cavity: epidemiology and aetiology. *Periodontol 2000* **57**, 19-37 (2011).
28. Mahdavi Fard, A. & Pourafkari, L. Images in clinical medicine. The hairy eyeball--limbal dermoid. *N Engl J Med* **368**, 64 (2013).
29. Balland, O. *et al.* Canine bilateral conjuntivo-palpebral dermoid: description of two clinical cases and discussion of the relevance of the terminology. *Case Rep Vet Med.*, **2015**, Article ID876141 (2015).
30. Onuigbo, W.I. & Ezegwui, I.R. Ophthalmic presentation of epidermoid cysts in an African community. *Int Ophthalmol* **24**, 279-281 (2001).
31. Nowell, C.S. *et al.* Chronic inflammation imposes aberrant cell fate in regenerating epithelia through mechanotransduction. *Nat Cell Biol* **18**, 168-180 (2016).
32. Mishra, D.K. *et al.* Differential Expression of Stem Cell Markers in Ocular Surface Squamous Neoplasia. *PLoS One* **11**, e0161800 (2016).
33. Fenger, C. & Filipe, M.I. Pathology of the anal glands with special reference to their mucin histochemistry. *Acta Pathol Microbiol Scand A* **85**, 273-285 (1977).
34. Roberts, J.M. *et al.* Papillary Immature Metaplasia of the Anal Canal: A Low-grade Lesion That Can Mimic a High-grade Lesion. *Am J Surg Pathol* **40**, 348-353 (2016).
35. Bilimoria, K.Y. *et al.* Squamous cell carcinoma of the anal canal: utilization and outcomes of recommended treatment in the United States. *Ann Surg Oncol* **15**, 1948-1958 (2008).
36. Brady, A. & McCluggage, W.G. Ectodermal structures within the uterine cervix and vagina: report of a series of cases. *Int J Gynecol Pathol* **32**, 602-605 (2013).
37. Kondi-Pafiti, A., Grapsa, D., Papakonstantinou, K., Kairi-Vassilatou, E. & Xasiakos, D. Vaginal cysts: a common pathologic entity revisited. *Clin Exp Obstet Gynecol* **35**, 41-44 (2008).
38. Slack, J.M. Homoeotic transformations in man: implications for the mechanism of embryonic development and for the organization of epithelia. *J Theor Biol* **114**, 463-490 (1985).
39. Hacker, N.F., Eifel, P.J. & van der Velden, J. Cancer of the vagina. *Int J Gynaecol Obstet* **131 Suppl 2**, S84-87 (2015).
40. Lee, S.H., Lee, D.J., Kim, K.M., Kim, K.N. & Kang, J.K. Ectopic sebaceous glands in the oesophagus: a case report and review of literature. *Scott Med J* **59**, e1-5 (2014).
41. Akiyama, S. *et al.* Esophageal cyst, a case report and a review of the literature. *Jpn J Surg* **10**, 338-342 (1980).

42. Venter, F.S. & Learmonth, G. Esophageal squamous metaplasia: nudging the esophageal carcinoma morphogenetic paradigm. *Med Hypotheses* **58**, 270-275 (2002).
43. Wang, X. *et al.* Residual embryonic cells as precursors of a Barrett's-like metaplasia. *Cell* **145**, 1023-1035 (2011).
44. Takubo, K. Squamous metaplasia with reserve cell hyperplasia in the esophagogastric junction zone. *Acta Pathol Jpn* **31**, 349-359 (1981).
45. Chiu, K.W., Wu, C.K., Lu, L.S., Eng, H.L. & Chiou, S.S. Diagnostic pitfall of sebaceous gland metaplasia of the esophagus. *World J Clin Cases* **2**, 311-315 (2014).
46. Smyth, E.C. *et al.* Oesophageal cancer. *Nat Rev Dis Primers* **3**, 17048 (2017).
47. Wolff, M., Rosai, J. & Wright, D.H. Sebaceous glands within the thymus: report of three cases. *Hum Pathol* **15**, 341-343 (1984).
48. Monaco, F., Barone, M. & Monaco, M. Intrathymic epidermoid cyst: a very rare condition. *Asian Cardiovasc Thorac Ann* **23**, 323-324 (2015).
49. Luo, Y., Li, J.L., Yang, L. & Zhang, W. Chemotherapy with gemcitabine plus cisplatin in patients with advanced thymic squamous cell carcinoma: Evaluation of efficacy and toxicity. *Thorac Cancer* **7**, 167-172 (2016).
50. Wang, W., Lv, W. & Tian, Y. Epidermoid cyst of the urinary bladder: a rare case. *Urol J* **11**, 1502-1503 (2014).
51. Liang, F.X. *et al.* Cellular basis of urothelial squamous metaplasia: roles of lineage heterogeneity and cell replacement. *J Cell Biol* **171**, 835-844 (2005).
52. Shokeir, A.A. Squamous cell carcinoma of the bladder: pathology, diagnosis and treatment. *BJU Int* **93**, 216-220 (2004).
53. Uthmann, U. & Terhorst, B. Dermoid cyst of the prostate with contralateral renal agenesis. *Br J Urol* **53**, 479 (1981).
54. Lager, D.J., Goeken, J.A., Kemp, J.D. & Robinson, R.A. Squamous metaplasia of the prostate. An immunohistochemical study. *Am J Clin Pathol* **90**, 597-601 (1988).
55. Malik, R.D. *et al.* Squamous cell carcinoma of the prostate. *Rev Urol* **13**, 56-60 (2011).
56. Nettesheim, P. & Martin, D.H. Appearance of glandlike structures in the tracheobronchial tree of aging mice. *J Natl Cancer Inst* **44**, 687-693 (1970).
57. Jetten, A.M., Vollberg, T.M. & Nervi, C. Hyperplasia and squamous metaplasia in the tracheobronchial epithelium: alterations in the balance of growth and differentiation factors. *Adv Exp Med Biol* **320**, 89-93 (1992).
58. Junker, K. Pathology of tracheal tumors. *Thorac Surg Clin* **24**, 7-11 (2014).
59. Ishizaki, H., Iida, S., Koga, H., Shimamatsu, K. & Matsuoka, K. Epidermoid cyst of the ureter: a case report. *Int J Urol* **14**, 443-444 (2007).
60. Hoshi, A. *et al.* [A case of squamous metaplasia of the ureter]. *Hinyokika Kiyo* **50**, 207-209 (2004).
61. Holmang, S., Lele, S.M. & Johansson, S.L. Squamous cell carcinoma of the renal pelvis and ureter: incidence, symptoms, treatment and outcome. *J Urol* **178**, 51-56 (2007).
62. Battal, M., Bostanci, O., Basak, T., Kartal, K. & Ekiz, F. Pure squamous cell carcinoma of the duodenum. *Case Rep Surg* **2015**, 714640 (2015).
