# Supplementary material for: Tp63-expressing adult epithelial stem cells cross lineages boundaries revealing latent hairy skin competence
Source: Nat Commun. 2020 Nov 6;11:5645. doi: 10.1038/s41467-020-19485-3 (PMC7648065; doi:10.1038/s41467-020-19485-3)
Supplement: Supplementary file 3 — Reporting Summary [file 41467_2020_19485_MOESM3_ESM.pdf]

nature research

Corresponding author(s): Y. Barrandon
Last updated by author(s): Sep 15, 2020

Reporting Summary

Nature Research wishes to improve the reproducibility of the work that we publish. This form provides structure for consistency and transparency in reporting. For further information on Nature Research policies, see our Editorial Policies and the Editorial Policy Checklist.

Statistics

For all statistical analyses, confirm that the following items are present in the figure legend, table legend, main text, or Methods section.

n/a	Confirmed	
☐	☒	The exact sample size (*n*) for each experimental group/condition, given as a discrete number and unit of measurement
☐	☒	A statement on whether measurements were taken from distinct samples or whether the same sample was measured repeatedly
☐	☒	The statistical test(s) used AND whether they are one- or two-sided<br>*Only common tests should be described solely by name; describe more complex techniques in the Methods section.*
☒	☐	A description of all covariates tested
☒	☐	A description of any assumptions or corrections, such as tests of normality and adjustment for multiple comparisons
☐	☒	A full description of the statistical parameters including central tendency (e.g. means) or other basic estimates (e.g. regression coefficient) AND variation (e.g. standard deviation) or associated estimates of uncertainty (e.g. confidence intervals)
☐	☒	For null hypothesis testing, the test statistic (e.g. *F*, *t*, *r*) with confidence intervals, effect sizes, degrees of freedom and *P* value noted<br>*Give P values as exact values whenever suitable.*
☒	☐	For Bayesian analysis, information on the choice of priors and Markov chain Monte Carlo settings
☒	☐	For hierarchical and complex designs, identification of the appropriate level for tests and full reporting of outcomes
☒	☐	Estimates of effect sizes (e.g. Cohen's *d*, Pearson's *r*), indicating how they were calculated

Our web collection on statistics for biologists contains articles on many of the points above.

Software and code

Policy information about availability of computer code

Data collection	Microscopy: Axiovision Rel. version 4.6 or 4.8 (Zeiss)<br>Macroscopy:Axiovision Rel. version 4.6 (Zeiss)<br>FACS: FACSDiva version 8.0.1 Becton Dickinson<br>RNAseq: Illumina Pipeline Software version 1.82<br>Karyotype: Smartcapture VP software (version not provided by Chrombios GmbH)<br>qPCR: Quantstudio qPCR 6 software, version 1.2.<br>Microarray sequencing: Illumina Pipeline Software version 1.82
Data analysis	Microscopy: Adobe Photoshop CS4 and version 21.1.3. ImageJ/Fiji version 2.0.0 and anterior<br>Macroscopy: Adobe Photoshop CS4<br>RNAseq: Ensembl, v. Rnor version 6.0.87, RSeQC version 2.3.7, R versions 3.3.2 and 3.6.2, EdgeR package, version 3.14.0, Limma package version 3.28.21, Cutadapt version 1.8, Fastq_screen version 0.9.3, Reaper version 15-065, Assembly, version Rnor 6.0, STAR version 2.5.2b, Htseq-count version 0.6.1, Gorilla (Eden et al., 2009, http://cbl-gorilla.cs.technion.ac.il), Vennerable package (version 3) in R, microsoft excel version 16.16.23.<br>FACS: FACSDiva version 8.0.1 Becton Dickinson<br>Microarray: Bioconductor Limma package, microsoft excel version 16.16.23. DNA-Chip Analyzer (dChip) (http://biosun1.harvard.edu/~cli/dchip.exe).<br>Karyotype: Quips (Vysis), SmartCapture VP software (Digital Scientific, Cambridge) (versions not provided by Chrombios GmbH).<br>qPCR: Expression suite v.1.0.4 (Applied Biosystems), GraphPad prism v7 and v8.4.3

For manuscripts utilizing custom algorithms or software that are central to the research but not yet described in published literature, software must be made available to editors and reviewers. We strongly encourage code deposition in a community repository (e.g. GitHub). See the Nature Research guidelines for submitting code & software for further information.

Data

Policy information about availability of data

All manuscripts must include a data availability statement. This statement should provide the following information, where applicable:

- Accession codes, unique identifiers, or web links for publicly available datasets
- A list of figures that have associated raw data
- A description of any restrictions on data availability

Microarray and RNAseq have deposited in the GEO database (Gene Expression Omnibus, https://www.ncbi.nlm.nih.gov/geo/) under the accession codes GSE116717 and GSE116719 .
Previously published microarray data were used in this article and are referenced under the GEO number GSE21686 (Bonfanti et al., 2010).
Figures associated to raw datas: figure 3, supplementary figure 6, supplementary table 3, supplementary data 1 to 3.
All data are available within this article, the supplementary information and are available upon request from the corresponding authors, Y.B or S.C.

Field-specific reporting

Please select the one below that is the best fit for your research. If you are not sure, read the appropriate sections before making your selection.

[x] Life sciences [ ] Behavioural & social sciences [ ] Ecological, evolutionary & environmental sciences

For a reference copy of the document with all sections, see nature.com/documents/nr-reporting-summary-flat.pdf

Life sciences study design

All studies must disclose on these points even when the disclosure is negative.

Sample size	No statistical methods were performed to determine sample size.<br>For In vivo experiments:<br>-Cell transplantations onto 2 to 20 independant mice for each cell type. 22 cell types were tested. The sample size is similar to previous publications (Claudinot et al., 2005; Bonfanti et al., 2010).<br>- Polyploidy study, transplantations were performed onto 4 to 56 independant mice for each cell type. 1 to 17 subclones from 5 different cell types were tested. Among these 5 cell types, 4 were aneuploid and one was euploid (control of engraftment).<br>- Tissue transplantation, 2 to 39 independant mice were transplanted for each tissue type. 18 tissue types were tested. The sample size is similar to a previous publication (Oshima et al., 2001).<br>For in vitro experiments:<br>- Microarray, we used 2 biological replicates for each cell type. Sample size was chosen based on our prior studies using the same types of assays, which were published previously (Bonfanti et al., 2010).<br>- qPCR: 3 technical replicates for each cell type (17 cell types).<br>- Karyotype: 10 cells were analyzed per sample (Protocol fixed by ChromBios GmbH).
Data exclusions	No data exclusion
Replication	Cell transplantation, Tissue transplantation and polyploidy experiments: Each mouse is considered as an independant experiment.<br>Efficiency is various and is indicated in supplementary figure 3, supplementary figure 4 and supplementary table 9. No transplantation session (several cell types the same day) were totally unsuccessful.
Randomization	In vivo experiments: Each cell or tissue type was transplanted in a random order. The mouse ID number was then used to identify each sample. Animals were randomly distributed in the cages and biopsies were treated according to the ID number. Correspondance between ID and cell type was performed before the histology procedure.<br>qPCR: the 17 samples were analyzed in triplicates. According to the number of primers, cell samples were randomly allocated to PCR plates, with the same positive and negative samples (controls) on each multiwell. They were analyzed on 384-multiwells by groups of 7 samples, the distribution being performed randomly by a Hamilton microlab star robot. Normalization was performed with all the datas of the positive control present on each qPCR plaque. 3 housekeeping genes (tubb, Sdha, Tbp) were used.
Blinding	No blinding: the same experimentator performed several different parts of the work. To maximize the objectivity, samples were treated according to the mouse ID number for in vivo experiments.

Reporting for specific materials, systems and methods

We require information from authors about some types of materials, experimental systems and methods used in many studies. Here, indicate whether each material, system or method listed is relevant to your study. If you are not sure if a list item applies to your research, read the appropriate section before selecting a response.

Materials & experimental systems

n/a	Involved in the study
	☒ Antibodies
	☒ Eukaryotic cell lines
☒	Palaeontology and archaeology
	☒ Animals and other organisms
☒	Human research participants
☒	Clinical data
☒	Dual use research of concern

Methods

n/a	Involved in the study
☒	ChIP-seq
	☒ Flow cytometry
☒	MRI-based neuroimaging

Antibodies

Antibodies used

Primary antibodies:
1. Mouse anti-cytokeratin 4, clone 6B10, 1:200, #NCL-CK4, Novocastra
2. Rabbit anti-cytokeratin 5, 1:1000, #ab24647, Abcam.
3. Guinea Pig anti-cytokeratin 8/18, 1:100, #GP-11, Progen
4. Rabbit anti -cytokeratin 14: 1:1000, #PRB-155P-100, Covance
5. Mouse anti-cytokeratin 15, 1:100, #: MA5-11344, ThermoFisher
6. Guinea Pig anti-cytokeratin 31, 1:200, #GP-HHA1 , Progen
7. rabbit anti-DeltaNp63, 1:500, #619001, Biolegend
8. Rabbit anti-DsRed, 1:100 , # GTX59862, Genetex
9. Rabbit anti-filaggrin, 1:400 , #PRB-417P, Covance
10. Chicken anti-GFP, 1: 400, # ab13970, Abcam
11. Mouse anti-involucrin clone SY5, 1: 100, #MS-126-P1, ThermoFisher
12. Mouse anti-Ki67, clone B56,1:200 , BD Pharmingen, #556003
13. Mouse anti-LEKTI clone 1C11G6, 1:100, #39-0500, Invitrogen
14 Rabbit anti-Lhx2, 1: 500, #ab184337, Abcam.
15. Rabbit anti-loricrin, 1:200, Genetex, #GTX 116013
16. Rabbit anti-sox9, 1:500, #ab185966, Abcam
17. mouse anti-Tp63, clone 4A4, 1:500, Dako (#M7247) ou Genetex (#GTX23239) ou Abcam (#ab735)

Secondary antibodies:
1. Goat anti-mouse Alexa 568, 1:500, #A11004, Molecular Probes
2. Goat anti-rabbit Alexa568, 1:500, #A11011, Molecular Probes.
3. goat anti-mouse Alexa568, 1:500, #A11031, Molecular Probes.
3. goat anti-Chicken Alexa488, 1:500, #A11039, Molecular Probes.
4. Goat anti-Guinea Pig Alexa568, 1:500, #A11075. Molecular Probes.
5. Goat anti-Chicken Alexan 488, 1:500, #ab150169, Abcam.

Validation

All the following antibodies were validated on rat tissue sections before using in the experiments and were citated in the litterature.

1. Mouse anti-cytokeratin 4: https://www.leicabiosystems.com/fileadmin/img_uploads/novocastra_reagents/Novocastra_datasheets/ck4.pdf. Qi et al., 2010. Potential localization of putative stem/progenitor cells in human bulbar conjunctival epithelium.J. Cell.Physiol.
2. Rabbit anti-cytokeratin 5: Su et al., 2009. TApo63 prevents premature aging by promoting stem cell maintenance. Cell Stem Cell.
3. Guinea Pig anti-cytokeratin 8/18: https://www.progen.com/anti-keratin-k8-k18-guinea-pig-polyclonal-serum.html. Li et al. 2009. Fluorescence and Electron Microscopic Localization of F-actin in the Ependymocytes. J. Histochem. Cytochem. Histochem Cytochem.
4. Rabbit anti -cytokeratin 14: Wend et al., 2013. Wnt/beta-catenin signalling induces MLL to create epigenetic changes in salivary gland tumours. EMBO J.
5. Mouse anti-cytokeratin 15: https://www.thermofisher.com/antibody/product/Cytokeratin-15-Antibody-clone-LHK15-Monoclonal/MA5-11344. Amoh et al., 2005. Multipotent nestin-positive, keratin-negative hair-follicle bulge stem cells can form neurons. Proc. Natl. Acad. Sci.
6. Guinea Pig anti-cytokeratin 31: https://www.progen.com/anti-acidic-hair-keratin-k31-guinea-pig-polyclonal-serum.html. Kretzschmar et al., 2014. BLIMP1 is required for postnatal epidermal homeostasis but does not define a sebaceous gland progenitor under steady-state conditions. Stem cell reports.
7. rabbit anti-DeltaNp63: https://www.biolegend.com/en-us/products/purified-anti-p63-deltan-antibody-2179. Medawar et al., 2008. DeltaNp63 is essential for epidermal commitment of embryonic stem cells. PLoS One.
8. Rabbit anti-DsRed: http://www.genetex.com/RFP-dsRed-antibody-GTX59862.html. Liu et al., 2012. Coordinate lentiviral expression of Cre recombinase and RFP/EGFP mediated by FMDV 2A and analysis of Cre activity. J. Cell. Biochem.
9. Rabbit anti-filaggrin: Wallace et al., 2012. Deletion of K1/K10 does not impair epidermal stratification but affects desmosomal structure and nuclear integrity. J. Cell Sci.
10. Chicken anti-GFP: http://www.abcam.com/gfp-antibody-ab13970.html. Shan Z et al., 2018. Basal condensation of Numb and Pon complex via phase transition during Drosophila neuroblast asymmetric division. Nat Commun.
11. Mouse anti-involucrin: https://www.thermofisher.com/order/catalog/product/MS-126-P1ABX. Bonfanti et al., 2010. Microenvironmental reprogramming of thymic epithelial cells to skin multipotent stem cells. Nature.

12. Mouse anti-Ki67: Vitte et al., 2017. Timing of Smarcb1 and Nf2 inactivation determines schwannoma versus rhabdoid tumor development. Nature Comm.
13. Mouse anti-LEKTI: https://tools.thermofisher.com/content/sfs/manuals/390500_Rev0609.pdf. Leusink et al., 2015. The Co-Expression of Kallikrein 5 and Kallikrein 7 Associates with Poor Survival in Non-HPV Oral Squamous-Cell Carcinoma. Pathobiol.
14 Rabbit anti-Lhx2: https://www.abcam.com/lhx2lh2-antibody-epr20449-ab184337.html. Liu B et al., 2018. Forced Expression of Foxg1 in the Cortical Hem Leads to the Transformation of Cajal-Retzius Cells into Dentate Granule Neurons. J Dev Biol.
15. Rabbit anti-loricrin: Qian et al. LncRNA expression profile of ΔNp63α in cervical squamous cancers and its suppressive effects on LIF expression. Cytokine.
16. Rabbit anti-sox9: https://www.abcam.com/sox9-antibody-epr14335-78-ab185966.html. Nguyen et al. , 2018. FGF signalling controls the specification of hair placode-derived SOX9 positive progenitors to Merkel cells. Nat Commun.
17. mouse anti-Tp63: Senoo et al., 2007. p63 Is Essential for the Proliferative Potential of Stem Cells in Stratified Epithelia. Cell.

Secondary antibodies (data from citeab.com)
1. Goat anti-mouse Alexa 568: 969 citations
2. Goat anti-rabbit Alexa568: 1149 citations
3. goat anti-mouse Alexa568: 758 citations
3. goat anti-Chicken Alexa488: 856 citations
4. Goat anti-Guinea Pig Alexa568: 114 citations
5. Goat anti-Chicken Alexa488: 62 citations

Eukaryotic cell lines

Policy information about cell lines

Cell line source(s)	3T3-J2 were from the Howard Green Lab, Department of Cellular and Molecular Physiology, Harvard Medical School, Boston, Massachusetts 02115 USA, (private gift). Reference: Rheinwald and Green, 1975. Cell; Barrandon et Green, PNAS, 1987; Gallico et al., NEJM, 1984.
Authentication	No molecular identification methods were used . Cells are kept in liquid nitrogen in labelled amps and located according to a specific listing.
Mycoplasma contamination	negative for mycoplasma
Commonly misidentified lines (See ICLAC register)	No commonly misidentified lines were used in this study

Animals and other organisms

Policy information about studies involving animals; ARRIVE guidelines recommended for reporting animal research

Laboratory animals	- EGFP rats: “CZ-004” SDTgN (act-EGFP)obsCZ-004: Japan SLC (Hamamatsu, Japan) (Ito et al., 2001). Tissues were dissected from males and females at postnatal day 9 to 201.<br>- Athymic (Swiss Nude-/-) mice: Charles River Breeding Laboratories (Les Oncins, France). Transplantations were performed on female athymic mice over 6 weeks-old.<br>- OF1 mice: Charles River Breeding Laboratories (Les Oncins, France): female and male pups were used at post-natal day 0 to 2.<br>- Rosa26 mice (B6;129S-Gt(ROSA)26Sor/J; JAX 002073; Friedrich and Soriano, 1991): Jackson Laboratories (USA). Males and females over 3 weeks-old.<br>- Lgr5 mice (B6.129P2-Lgr5<tm1(cre/ERT2)Cle>/J, JAX 008875; Barker et al., 2007) : Jackson Laboratories (USA). Males and females over 3 weeks-old.<br>- DsRed (B6.Cg-Tg(CAG-DsRed*MST)1Nagy/J, JAX 006051; Vintersten et al., 2004) : gifted by C. Brisken (EPFL, CH). Males and females over 3 weeks-old.<br>- Lef1 mice: (B6-Lef1tm1Rug, Van Genderen C, et al., 1994): gifted by R. Grosschedl (Freiburg, Germany). Female and male pups were used at post-natal day 0 to 2.
Wild animals	No wild animals were used
Field-collected samples	No field-collected samples were used
Ethics oversight	All animal experiments were authorized by the veterinary commission of the Office of the Canton de Vaud (SCAV, Switzerland) under licence numbers 1525, 1790, 2369, 2370, 2855 and 3294, without any opposition of the federal veterinary office (see animal chapter methods). Experiments were performed according to the Swiss legislation and the European Community Council Directive (86/609/EEC)

Note that full information on the approval of the study protocol must also be provided in the manuscript.

Flow Cytometry

Plots

Confirm that:

- [x] The axis labels state the marker and fluorochrome used (e.g. CD4-FITC).
- [x] The axis scales are clearly visible. Include numbers along axes only for bottom left plot of group (a 'group' is an analysis of identical markers).
- [x] All plots are contour plots with outliers or pseudocolor plots.
- [x] A numerical value for number of cells or percentage (with statistics) is provided.

Methodology

Sample preparation	EGFP rat keratinocytes were cultured on a feeder layer of lethally irradiated 3T3-J2 cells (Rheinwald and Green, 1975), in cFAD medium (mixture 3:1 of Dulbecco Modified Eagle's Medium and Ham's F12), supplemented by 10% Fetal Calf serum, 10E-6M cholera toxin, 2.10E-9M 3,3',5-triiodo-l-thyronin (T3), 5 ug.ml-1 insulin and 0.4 ug.ml-1 hydrocortisone . Cells were fed every 3-4 days with supplemented cFAD containing 1 ng.ml-1 of human recombinant EGF. At day 5 or 6, cells were trypsinized with 0.05% trypsin-0.1% EDTA, filtered through a 70 um cell-strainer and centrifuged. They were resuspended in 1X HBSS-2%FCS-0.01% EDTA-20mM HEPES containing DAPI.
Instrument	FACS Aria SORP (BD Biosciences) Serial N° : P5H400001<br>FACSFusion SORP (BD Biosciences) Serial N°: P65828273001
Software	FACSDiva version 8.0.1 Becton Dickinson
Cell population abundance	Count were performed by the FACSDiva software. Depending on the samples, 10-73% total cells were considered as GFP-positive and alive.
Gating strategy	A suspension of cultured EGFP-rat keratinocytes were gated to select alive GFP-positive cells.<br>FSC-A/SSC-a was first used to remove debris, then FSC-W/FSC-H and SSC-W/SSC-H were used to discard doublets and keep singlets. GFP499/DAPI355 was then used to to select alive cells according to the DAPI dye exclusion, and GFP-positive cells according to the GFP expression. Strategy is represented in Supplementary figure 7.

- [x] Tick this box to confirm that a figure exemplifying the gating strategy is provided in the Supplementary Information.
