# Supplementary material for: Tp63-expressing adult epithelial stem cells cross lineages boundaries revealing latent hairy skin competence
Source: Nat Commun. 2020 Nov 6;11:5645. doi: 10.1038/s41467-020-19485-3 (PMC7648065; doi:10.1038/s41467-020-19485-3)
Supplement: Supplementary file 4 — Description of Additional Supplementary Files [file 41467_2020_19485_MOESM4_ESM.pdf]

Description of Additional Supplementary Files

Supplementary Data 1. List of the up- and down-regulated genes in the microarray analysis. Gene expression in different non-hairy Tp63-expressing epithelial stem cells cultured before and after transplantation into the skin of newborn wild type mouse compared to that of multipotent stem cells of the whisker follicle. Microarray analysis. Fold change > 2, adjusted p-value <0.05. NS: non-significant.

Supplementary Data 2. List of the up- and down-regulated genes in the RNA-Seq analysis. Gene expression in different non-hairy Tp63-expressing epithelial stem cells cultured before and after transplantation into the skin of newborn wild type mouse compared to that of multipotent stem cells of the whisker follicle. RNA-Seq analysis. Fold change > 2, adjusted p-value <0.05. NS: non-significant.

Supplementary Data 3. List of the genes in common in the RNA-Seq analysis. Up- and down-regulated genes in common in different non-hairy Tp63-expressing epithelial stem cells cultured before and after transplantation into the skin of newborn wild type mouse compared to that of multipotent stem cells of the whisker follicle. RNA-Seq analysis. Fold change > 2, adjusted p-value <0.05.
